# Postoperative Delirium after elective and emergency surgery: analysis and checking of risk factors. A study protocol

**DOI:** 10.1186/1471-2482-5-12

**Published:** 2005-05-28

**Authors:** Vanni Agnoletti, Luca Ansaloni, Fausto Catena, Rabbih Chattat, Angelo De Cataldis, Gianfranco Di Nino, Claudio Franceschi, Stefano Gagliardi, Rita Maria Melotti, Antonella Potalivo, Mario Taffurelli

**Affiliations:** 1 Department of Anaesthesiology, "S. Orsola-Malpighi" Hospital, University of Bologna, Italy; 2 Department of Emergency Surgery, S. Orsola-MalpighiHospital, University of Bologna, Italy; 3 Department of Psychology, University of Bologna, Italy; 4 Department of Experimental Pathology, University of Bologna, Italy

## Abstract

**Background:**

Delirum is common in hospitalized elderly patients and may be associated with increased morbidity, length of stay and patient care costs. Delirium (acute confusional state) is defined as an acute disorder of attention and cognition. In elderly patients, delirium is often an early indicator of patho-physiological disturbances. Despite landmark studies dating back to the 1940s, the pathogenesis of Delirium remains poorly understood. Early investigators noted that Delirium was characterized by global cortical dysfunction that was associated predominantly with specific electroencephalographic changes. It's important to understand the risk factors and incidence of Delirium. Some of the risk factors are already identified in literature and can be summarized in the word "VINDICATE" which stands for: Vascular, Infections, Nutrition, Drugs, Injury, Cardiac, Autoimmune, Tumors, Endocrine. Aims of this study are: to re-evaluate the above mentioned clinical risk factors, adding some others selected from literature, and to test, as risk factors, a pattern of some genes associated to cognitive dysfunction and inflammation possibly related to postoperative Delirium.

**Design:**

All patients admitted to our Emergency Unit who are meet our inclusion/exclusion criteria will be recruited. The arising of postoperative Delirium will select incidentally two groups (Delirium/non Delirium) and the forward analysis of correlate risk factors will be performed. As in a typical observational case/control study we will consider all the exposure factors to which our population are submitted towards the outcome (presence of Delirium). Our exposures are the following: ASA, Pain (SVS; VAS), Blood gas analysis (pH; Hb; pO2; pCO2), Residence pharmacological therapy (BDZ; hypnotics; narcotic drugs; alcohol; nitrous derivates), Body temperature, Arterial pressure, Heart frequency, Breath frequency, Na, K, Creatinin, Glicemia, Albumin, Hct, White blood cells, Glasgow Coma Scale (GCS), Cognitive state (SPMSQ), Functional state (ADL and IADL), Psychological Distress (HADS), Cumulative Illness Rating Scale (CIRS), Hypotension (classified in: light; moderate and severe and duration), Blood loss (classified in: < 2 lt and > 2 lt), Blood transfusions (< 2 lt and > 2 lt), Quantity of red cells and plasma transfusions, Visual VAS / SVS (timing: I-II-III post-operative day), Red cells and Plasma transfusions, Blood count evaluation and Saturation (O_2_%), Postoperative analgesia (Emilia-Romagna protocol), Presence of malignant tumoral disease, APACHE Score II. Moreover the presence of some relevant genetic polymorphisms will be studied in different genes such as IL-6, IL-10, TNF-alpha, and IL-1 cluster.

## Background

Delirum is common in hospitalized elderly patients and may be associated with increased morbidity[1], length of stay and patient care costs[2]. The classic manifestations of this syndrome are impaired cognition and decreased ability to maintain attention[3]. Efforts to understand this syndrome require a thorough understanding of its causes and the ability to predict who is at risk. Each year Delirium complicates hospital stays for more than 2.3 milion older people, involves more than 17.5 million inpatients days, and accounts for more than $4 billion of Medicare expenditure[4]. Substantial additional costs accrue after discharge from the hospital, because of the increased need for institutionalazisation, rehabilitation, and home care[5].

Delirium (acute confusional state) is defined as an acute disorder of attention and cognition[6].

It represents a syndrome of disruption of one's state of consciousness, concentration, perception, memory, cognition, orientation and psychomotor behavior. The most prominent symptoms among these are the inability to concentrate and changes in the state of alertness[7].

The reported incidence of Delirium in acutely ill elderly patients during hospitalization ranges from 7 to 61.3% in the US depending on the population studied and the criteria used for diagnosis[8]. In elderly patients, delirium is often an early indicator of patho-physiological disturbances.

It's important to understand the risk factors and incidence of Delirium, because we agree with the Inouye's model of the cumulative effects of baseline vulnerability factors and precipitating factors for Delirium. Baseline vulnerability factors are defined as predisposing factors for Delirium present upon the admission. Precipitating factors are defined as noxious insults or hospitalization-related factors that contribute to Delirium[9].

Despite landmark studies dating back to the 1940s, the pathogenesis of Delirium remains poorly understood. Early investigators noted that Delirium was characterized by global cortical dysfunction that was associated predominantly with specific electroencephalographic changes. These findings suggest an abnormality on a biochemical and electrophysiological level.

Although Delirium can develop at any time during hospitalization, it typically presents early in the postoperative period. A good preoperative evaluation should include a formal cognitive assessment in patients at risk of developing Delirium. Delirium usually persists for hours to days and can fluctuate throughout the course of the day. Although several formal cognitive test are useful in identifying Delirium, including the Mini-Mental-Status-Exam (MMSE) [10], the Confusion-Assessment-Method (CAM) and the Delirium Writing, as the MMSE can't distinguish Delirium from dementia, the CAM remains the most suitable test to check the presence of Delirium.

Some of the risk factors are already identified in literature and can be summarized in the word "VINDICATE" which stands for: Vascular, Infections, Nutrition, Drugs, Injury, Cardiac, Autoimmune, Tumors, Endocrine. During last year in the Emergency Surgery Unit of S. Orsola-Malpighi Hospital (Bologna, Italy) a pilot study was performed with the aim of checking and analyzing some of the most important risk factors for postoperative Delirium by using an observational case/control study. A series of 100 over 65 years old patients, admitted for either emergency or elective surgery, were included and submitted to a psychometric, clinical and biochemical assessment. Among all risk factors examined the following were statistically significantly related to postoperative Delirium:

• Origin from home or other medical garrisons;

• Cardiovascular pathology;

• Metabolic diseases (hypo-hyper-tyroidism; glycemic disturbances)

• ECG abnormalities (ECG);

• Intraoperative hypotension;

• Preoperative low level of hemoglobin;

• Preoperative low level fo hematocritus;

• Postoperative hypoglicemia;

• Use of nitrous derivate during anesthesia;

• Use of anticolynergic drugs during surgery;

• Blood and plasma transfusion during surgery;

• Mini Mental Status (MMS);

• ADL (Activity Day Living).

The aims of the present study are the followings:

• to re-evaluate the above mentioned clinical risk factors, adding some others selected from the literature.

• to test, as risk factors, a pattern of some genes associated to cognitive dysfunction and inflammation possibly related to postoperative Delirium.

## Methods

### Design

Observational analytical case/control study (outcome-exposure).

All patients admitted to our Emergency Unit who meet our inclusion/exclusion criteria will be recruited. The arising of postoperative Delirium will select incidentally two groups (Delirium/non Delirium) and the forward analysis of correlate risk factors will be performed.

As in a typical observational case/control study we will consider all the exposure factors to which our population are submitted towards the outcome (presence of Delirium).

Our exposures are the following:

#### 1) *Preoperative*

• ASA

• Pain (SVS; VAS)

• Blood gas analysis (pH; Hb; pO2; pCO2)

• Residence pharmacological therapy (BDZ; hypnotics; narcotic drugs; alcohol; nitrous derivates)

• Body temperature

• Arterial pressure

• Heart frequency

• Breath frequency

• Na; K; Creatinin; Glicemia; Albumin

• Hct

• White blood cells

• Glasgow Coma Scale (GCS)

• Cognitive state (SPMSQ)

• Functional state (ADL and IADL)

• Psychological Distress (HADS)

• Cumulative Illness Rating Scale (CIRS) (classified in 4 different steps):

- heart;

- hypertension;

- cardiovascular diseases;

- respiratory tract diseases;

- othorinolaryngoiatry diseases;

- digestive tract diseases (divided in to two tracts: proximal and distal);

- liver diseases;

- kidney diseases;

- urogenital diseases;

- bone and muscle diseases;

- neurological diseases;

- endocrino-metabolic diseases;

- mind status and behavior;

#### 2) *Intraoperative*

• Hypotension (classified in: light; moderate and severe and duration);

• Blood loss (classified in: < 2 lt and > 2 lt);

• Blood transfusions (< 2 lt and > 2 lt);

• Quantity of red cells and plasma transfusions.

#### 3) *Postoperative*

• VAS / SVS (timing: I-II-III post-operative day);

• Red cells and Plasma transfusions;

• Blood count evaluation and Saturation (O_2_%);

• Postoperative analgesia (Emilia-Romagna protocol);

• Presence of malignant tumoral disease;

• APACHE Score II

#### 4) *Genetic*

The presence of some relevant genetic polymorphisms will be studied in different genes such as IL-6, IL-10, TNF-alpha, and IL-1 cluster. The polymorphisms are the following :

1. -174 (C/G) at 5'-upstream of IL-6[15];

2. -1082 (G/A) at IL-10 promoter sequence[13],

3. -308 (G/A) at TNF-alpha promoter sequence[14],

4. -889 (C/T) in IL-1 alpha; -511 (C/T) in IL-1 beta; IL-1 Receptor Antagonist (86 bp repeated sequenze in introne 2)[11].

All these polymorphisms have an important role for immune response/inflammation pathways[12] and in this project their frequency will be correlated to postoperative Delirium insurgence. The products of these genes will be also measured in the plasma, together with cortisol levels, because the last is known to have an important role in Immune-Neuroendocrine System and in Delirium insurgence[16].

Blood samples (15–20 ml) from the enrolled patients at S. Orsola-Malpighi Hospital, at Department of Emergency Surgery, will be collected in tripotassium EDTA sterile tubes. These samples will be transported to the Laboratory of Immunology, directed by Prof Claudio Franceschi (Dipartimento di Patologia, Sperimentale, via S. Giacomo 12, Bologna). Plasma will be harvested as soon as possible and stored at -80°C, while the cell pellet will be stored separately at -80° for further DNA extraction. Genomic DNA extraction will be carried out by classical phenol/chloroform techniques according to standard procedures and then stored at -20°C. The genetic polymorphisms, will be studied according to the detailed protocol reported in above mentioned papers.

Briefly, genotyping analysis will be performed by means of PCR methods and gel electrophoresis analysis to test all genes, but IL-10 polymorphisms that will require a slight different method, named ARMS(Amplification Refractory Mutation System)-PCR, which is applied for single base biallelic polymorphism/mutation[13]. In particular, the study of single base biallelic polymorphism -1082 (C/A) at IL-10 promoter sequence will be analysed using the protocol here described. Two different 5' primers respectively specific for IL-10 -1082C and -1082A are separately mixed with a 3' primer in the following conditions: 200 ng of template DNA are mixed with a final concentration of 2 U of TaqGold-DNA polymerase, 200 mmol/L each of deoxynucleotyde and 1X reaction buffer and 0.5 mmol/L of each specific primer. Cycling is performed at 98°C for 12 min and 35 cycles at 96°C for 30, 54°C for 30, and 72°C for 30, followed by a final extension of 10 min at 72°C. PCR products are detected by electrophoresis on 2% agarose.

The plasma levels of gene products, i.e. the IL-6, IL-10, IL-1, TNF-alpha cytokines will be evaluated by Enzyme Linked ImmunoSorbent Assay (ELISA) standard procedures. Cortisol will be evalueted by usual biochemical-haematological assays.

DNA samples will be stored in the Department of Experimental Pathology, Section of Immunology, in the respect of the Italian current law related to privacy protection.

In case that some patients will decide to leave the study, DNA samples will be destroyed by hydrochloric acid pre-treatment and then disposed off in the biological refusals.

The presence of Delirium among patients enrolled in our study will be evaluated through CAM test interviews at the 1^st^, 2^nd^, 3^rd ^and 6^th ^postoperative day. To assess the severity of Delirium (previously diagnosed through CAM) the Delirium Rating Scale (DRS) will be used.

The follow-up of patients enrolled in this study will be completed by two others checks (at 3 and 6 months after the operation) in order to establish a possible correlation between the arising of postoperative Delirium and development of permanent cognitive dysfunctions and Dementia.

### Sample Size

The exact sample size will be 300 patients with a probable distribution of 1/4,26 in the two groups (Delirium/non Delirium), that has been calculated through our above mentioned pilot study. This study showed 19 Delirium patients over 100 included: for this reason we are expecting, among our population of 300 patients, 57 with postoperative Delirium.

### Power calculation

Sample size has been calculated to reach a confidence level of 95% with a power of 80% according to the results of the above mentioned pilot observational case/control study on 100 patients previously admitted to our Operative Unit. In this study 19 patients on 100 presented postoperative Delirium with a ratio, between two groups (81 not Delirium vs. 19 Delirium), of 4.26. Furthermore we selected MMSE with a cut off value of 7 as a variable considered as a risk factor in developing postoperative Delirium. Then we calculated the ratios of patients in the two groups with a value of MMSE below 7. This ratio resulted 0.26 for those who presented postoperative Delirium and 0.09 for the other group. So we calculated our sample size by a Proportion Difference Power/Sample Size Calculation using an online statistical calculator[17]. The calculated sample size was of 257 patients (49 Delirium and 208 Not Delirium). We expect to enrol 300 patients.

### Inclusion and exclusion criteria

Inclusion criteria are:

• Age > 65 years

Exclusion criteria

• Any condition preventing a correct evaluation of cognitive functions such as speech and sensory impairment or psychiatric disorders (language difficulties or psychiatric organic dysfunctions) which make difficult the administration of psychometric tests.

### Informed consent

In the informed consent form, patients will receive all the information about the study protocol, the confidential nature of personal data and will fill up a questionnaire before signing for acceptance or refusal, including the authorization for taking a sample of blood. All the medical information obtained from the patients will be kept confidentially among the research scientists conducting the study. In case of refusal to participate, there will be not any inconvenience to the patients. Patients will be free to withdraw from the study whenever they want without any obligation.

### Ethical approval

Approved by Ethical Committee of S. Orsola-Malpighi Hospital on March 1^st^, 2005

### Stopping rules

In our study we don't include the experimental use of any drug or surgical procedures, so we don't expect any inconvenience or complication.

### Primary endpoint

The evaluation of risk factors above mentioned and their power of correlation with incidence of Delirium.

The evaluation of Delirium presence as a possible risk factor in developing successive Dementia.

### Interim analysis

A preliminary statistical analysis will be performed after enrolling the first 150 patients.

### Data management – type of analysis – statistics test stated

All our data will be reported on a form on purpose created and subsequently collected in an appropriate computer database hold in the Emergency Surgery Unit.

After a preliminary descriptive analysis, univariate analysis will be performed by using Odds Ratio for categorical variables and T-test or U-Mann-Withney test for continuous variables depending on their distribution (Normal/Not normal) as appropriate. Multivariate analysis will be performed by using Logistic Regression (forward-conditional) introducing only variables estimated statistically significant in the univariate analysis. All statistical analysis will be performed using the statistical software SPSS 8.0 for Windows.

### Indemnities specified

No incentives are planned for the patients regarding the operation or the follow-up.

### Is the study clinically necessary?

Postoperative Delirium is one of the factors which influences morbidity and mortality after elective and emergency surgery and even engraves on economical budget of the Surgical Units.

Check and knowing the most important risk factors could help in establish prevention measures in order to reduce postoperative Delirium's incidence and could let us to manage it appropriately.

### Time frame – finishing date – reporting date

We expect to start the study next June. According to the number of patients admitted monthly in our Unit, the duration of the study can be approximately estimated of about one year.

## Competing interests

The author(s) declare that they have no competing interests.

## Authors' contributions

VA, LA, RC, AD, CF, AP, edited the manuscript

RMM, GD, MT drafted the manuscript

AD, SG performed the statistical analysis

All Authors participated in the design of the study.

All Authors read and approved the final manuscript.

## Pre-publication history

The pre-publication history for this paper can be accessed here:


